# A Novel *Deinococcus* Antioxidant Peptide Mitigates Oxidative Stress in Irradiated CHO-K1 Cells

**DOI:** 10.3390/microorganisms12112161

**Published:** 2024-10-26

**Authors:** Sangyong Lim, Ha-Yeon Song, Hae Ran Park, Ki Bum Ahn

**Affiliations:** 1 Radiation Biotechnology Division, Korea Atomic Energy Research Institute, Jeongeup 56212, Republic of Korea; hysong93@kaeri.re.kr (H.-Y.S.); ahnkb@kaeri.re.kr (K.B.A.); 2 Department of Radiation Science, University of Science and Technology, Daejeon 34113, Republic of Korea; 3 Cyclotron Applied Research Section, Korea Atomic Energy Research Institute, Jeongeup 56212, Republic of Korea; hrpark@kaeri.re.kr

**Keywords:** antioxidant peptide, *Deinococcus deserti*, radiation protection, leaderless mRNA

## Abstract

Reactive oxygen species (ROS), byproducts of cellular metabolism and environmental factors, are linked to diseases like cancer and aging. Antioxidant peptides (AOPs) have emerged as effective countermeasures against ROS-induced damage. The *Deinococcus* genus is well known for its extraordinary resilience to ionizing radiation (IR) and possesses complex antioxidant systems designed to neutralize ROS generated by IR. In this study, we developed four peptides, each containing 9 to 11 amino acids, from the leaderless mRNA (lmRNA) sequences of *D. deserti*. Lacking a 5′ untranslated region, lmRNAs directly initiate protein synthesis, potentially encoding small peptides such as AOPs. Of the four peptides, Ddes-P3 was found to exhibit significant antioxidant capabilities in vitro, effectively scavenging ABTS radicals. Ddes-P3 provided considerable defense against IR-induced oxidative stress in CHO-K1 cells, demonstrating a notable reduction in ROS production and lipid peroxidation. The peptide’s potential was highlighted by its ability to enhance cell survival and maintain mitochondrial membrane potential under irradiative stress, suggesting its utility as a nontoxic and effective radioprotector in mitigating radiation-induced cellular damage. This study explores the potential role of lmRNA in synthesizing AOPs within *Deinococcus*. Identifying lmRNAs that encode AOPs could deepen our understanding of their cellular resistance to oxidative stress and pave the way for creating innovative biotechnological and therapeutic AOPs.

## 1. Introduction

Reactive oxygen species (ROS), which are normal byproducts of cellular metabolism, can also arise from environmental exposures like radiation and certain chemicals. Excessive ROS accumulation can damage cells and is linked to diseases such as skin aging, neurodegeneration, and cancer [1,2]. Antioxidant peptides (AOPs) are active biochemicals that counteract reactive oxygen species (ROS) and protect cells from oxidative damage [1]. Some AOPs not only have potent antioxidant capabilities but also exhibit antibacterial, anti-inflammatory, and skin-healing properties [1,3]. These properties make them vital in diverse sectors, including food science, pharmaceuticals, and cosmetics [1,3,4]. Due to their immense genetic diversity, microorganisms can generate a broad spectrum of peptides. This diversity makes them essential resources for discovering and engineering novel AOPs that effectively combat oxidative stress and mitigate related cellular damage [5].

Ionizing radiation (IR) is a type of radiation energetic enough to ionize atoms or molecules by detaching electrons. In biological tissues, IR predominantly interacts with water, the most abundant cellular component, accounting for about 70% of cells. This interaction leads to water radiolysis, resulting in the formation of highly reactive species like hydroxyl radicals that cause extensive damage to essential biomolecules such as DNA, lipids, and proteins [6,7]. The *Deinococcus* genus is renowned for its extraordinary resistance to extreme environmental stresses, particularly ionizing radiation (IR). A representative species of this genus, *Deinococcus radiodurans* (*D. radiodurans*), is distinguished by its rapid and efficient DNA repair mechanisms [8]. In *D. radiodurans*, double-strand DNA breaks, the most lethal damage from IR, are repaired primarily through the homologous recombination (HR) pathway via extended synthesis-dependent strand annealing (ESDSA). This organism’s unique ESDSA mechanism is enhanced by unconventional HR proteins such as RecA and UvrD and *Deinococcus*-specific proteins likr DdrA and DdrB, setting it apart from typical organisms [9]. *D. radiodurans* can also withstand high levels of oxidative stress, which is attributed to its robust enzymatic and nonenzymatic antioxidative defense mechanisms [10].

Daly et al. [11] investigated the role of small molecules, particularly peptides, in protecting cellular proteins from oxidative damage. They found that manganese (Mn) in the *D. radiodurans* ultrafiltrates predominantly binds to small molecules (<3 kDa). The Mn complex, composed of manganese ions (Mn^2+^) bound to specific peptides and orthophosphate (Pi), forms intracellular antioxidant complexes. These complexes safeguard the bacterium by scavenging ROS and preventing oxidative damage caused by IR to cellular components [11,12].

A synthetic decapeptide, DEHGTAVMLK (DP1), was formulated to reflect the composition of some the most abundant amino acids found in *D. radiodurans* ultrafiltrates, known for their pronounced antioxidant properties [11]. When combined with Mn^2+^ and Pi, DP1 efficiently neutralizes ROS, acting as a potent antioxidant both in vivo and in vitro [13]. It grants extraordinary radiation resistance to enzymes, preserving their activity under extreme conditions, and protects cultured human cells and mice from severe damage caused by IR [11,14,15]. The DP1 complex has also been leveraged in developing radiation-inactivated whole-cell vaccines against several pathogens, such as poliovirus, methicillin-resistant *Staphylococcus aureus*, and multidrug-resistant *Acinetobacter baumannii*, due to its strong antioxidant properties, which stabilize and protect vaccine antigens [16,17,18,19].

Leaderless mRNA (lmRNA), also known as leaderless genes, are mRNA molecules that lack the 5′ untranslated region (5′-UTR) and Shine–Dalgarno sequence typically found in conventional mRNAs [20]. While lmRNAs are rare in enterobacteria like *Escherichia coli*, they are more prevalent in other bacteria such as *Deinococcus* species [21]. For instance, RNA sequencing has shown that 60% of the mRNAs in *D. deserti* are leaderless, with transcription start sites (TSSs) located precisely at or very close to the translation initiation codons (AUG or GUG). The proteomic analysis confirmed that these lmRNAs are efficiently translated, making substantial contributions to the proteome of *D. deserti* [22]. The study also identified 173 additional lmRNAs that may encode novel proteins and small peptides, potentially crucial for radiation tolerance by protecting proteins from oxidative damage [22]. In this study, we randomly selected four potential novel peptides from *D. deserti*, ranging from 9 to 11 amino acid (aa) residues, and examined their antioxidant activities.

## 2. Materials and Methods

### 2.1. Peptide Synthesis

Peptides were produced by Bionics (Seoul, Republic of Korea) and their purity, confirmed through RP-HPLC analysis, was at least 95%.

### 2.2. Analysis of the Physicochemical Characteristics of Synthetic Peptides

Online tools were utilized to predict the physicochemical properties of the synthetic peptides. The peptide property calculator at https://pepcalc.com/ (8 July 2024) estimated their net charge and water solubility. Additionally, the ProtParam tool at http://web.expasy.org/protparam/ (15 July 2024) facilitated the analysis of their isoelectric point (pI), molecular weight (MW), instability index, and grand average of hydropathicity (GRAVY).

### 2.3. ABTS Radical Scavenging Activity Assay

The ABTS (2,2′-azino-bis(3-ethylbenzothiazoline-6-sulphonic acid)) assay was performed following the modified protocol by Re et al. [23]. The working solution for the assay was prepared by mixing 7 mM ABTS with 2.45 mM potassium persulfate. Peptide samples were prepared in phosphate-buffered saline (PBS) and aliquoted into a 96-well plate at 10 µL per well. Alongside the samples, a negative control containing only PBS was also included to validate the assay results. Both sample and control wells received 150 µL of the ABTS working solution. The plate was incubated at room temperature for five minutes in the absence and presence of 1 mM MnCl_2_. After incubation, 100 µL of stop solution was introduced to each well to halt the reaction. Absorbance was measured at 760 nm using an Epoch 2 microplate reader (BioTek, Winooski, VT, USA). The percentage inhibition of ABTS radical scavenging activity was determined by comparing the absorbance values of the test samples to the negative control.

### 2.4. Cell Cultures and X-Ray Irradiation

Chinese Hamster Ovary (CHO)-K1 cell lines were obtained from the American Type Culture Collection (ATCC; Manassas, VA, USA). The cells were cultured in RPMI-1640 medium (Gibco, Waltham, MA, USA) with 10% fetal bovine serum (FBS, Biowest, Riverside, MO, USA) and antibiotics (100 U/mL penicillin and 0.1 mg/mL streptomycin) at 37 °C in a 5% CO_2_ incubator. For irradiation, the culture medium was replaced with phosphate-buffered saline (PBS), and cells were exposed to X-rays at doses of 2, 4, 6, or 8 Gy using a cabinet X-ray irradiator (Faxitron X-Ray Corp., Wheeling, IL, USA) set at 160 keV and 1 mA with a dose rate of 0.3 Gy/min. Before conducting the main experiment, 5 mm diameter alanine dosimeters (Bruker Instruments, Rheinstetten, Germany) were placed in the irradiator to verify dosing accuracy. Post-irradiation, the actual absorbed doses were measured using an electron paramagnetic resonance analyzer (e-scan™ alanine dosimeter reader, Bruker BioSpin GmbH, Rheinstetten, Germany), following the standards of ISO/ASTM 51607:2013. The measured doses were confirmed to be within 5% of the intended doses of 2, 4, 6, or 8 Gy. Following irradiation, the medium was replaced with fresh RPMI-1640 supplemented with 10% FBS and 1% penicillin/streptomycin.

### 2.5. Cytotoxic Activity Assay

The effect of synthetic peptides on cell viability was evaluated using the CCK8 assay, as previously described [24]. CHO-K1 cells were seeded in 96-well plates at a density of 3 × 10^3^ cells/mL in RPMI-1640 medium and incubated overnight at 37 °C. Following incubation, cells were treated with the Ddes-P3 peptide for 24 h. Post-treatment, 10 μL of CCK-8 solution was added to each well, and the cells were incubated for 2 h. Absorbance was recorded at 450 nm using an Epoch 2 microplate reader (BioTek).

### 2.6. Colony-Forming Assay (Clonogenic Assay)

A clonogenic assay was conducted as previously described [25]. CHO-K1 cells were plated in six-well plates at a density of 3 × 10^5^ cells/mL and treated with 30 µM of Ddes-P3, which was dissolved in dimethyl sulfoxide (DMSO). For control groups, cells were treated with either PBS or 0.3% DMSO. After incubation for two hours, the cells were exposed to X-ray at a rate of 0.3 Gy/min. Following irradiation, the cells were incubated for seven days, during which the medium was refreshed every three days. At the end of the incubation period, colonies were fixed with 70% methanol and stained with 0.5% crystal violet. Colonies containing 50 or more cells were counted as clonogenic cells. The plating efficacy (*PF*) and surviving fraction (*SF*) were calculated using standard equations.

$$PF=\frac{No.\;of\;colonies\;}{No.\;of\;seeding\;cells}\times 100\;\mathrm{and}\;SF=\frac{PF\;of\;test\;group}{PF\;of\;control\;group}$$

### 2.7. Measurement of Intracellular ROS

Intracellular ROS levels were measured using chloromethyl-2,7-dichlorofluorescin diacetate (DCFH-DA; Sigma-Aldrich, St. Louis, MO, USA) through flow cytometry with a MACSQuant Analyzer (Miltenyi Biotec, Bergisch Gladbach, Germany). CHO-K1 cells were incubated with or without 30 μM Ddes-P3 for 4 h. Subsequently, the cells were rinsed with PBS and incubated with 20 μM DCFH-DA at 37 °C in darkness for one hour. Post-incubation, the cells were harvested and subjected to 4 Gy X-ray irradiation. The cells were then immediately analyzed by flow cytometry, recording at least 10,000 events per sample.

### 2.8. Annexin V/Propidium Iodide (PI) Assay

CHO-K1 cells were seeded in a six-well plate at a density of 3 × 10^5^ cells/mL and pretreated with either 10 or 30 μM MP-D3 for 3 h before X-ray irradiation. Two control groups were established: the nonirradiated group received a PBS treatment and the irradiated group received a 0.3% DMSO treatment. Post 24 h irradiation, the cells were collected using 0.25% trypsin-EDTA (Gibco), rinsed with PBS, and stained according to the manufacturer’s instructions using the Annexin V/PI apoptosis detection kit (BD Biosciences, San Jose, CA, USA). Stained cells were subjected to flow cytometry analysis using a MACSQuant Analyzer (Miltenyi Biotec). Data were subsequently processed and visualized with FlowJo V10 software (TreeStar, Ashland, OR, USA).

### 2.9. Measurement of Intracellular Malondialdehyde (MDA) Level

Lipid peroxidation was assessed by quantifying MDA using a colorimetric MDA assay kit from Abcam (Waltham, MA, USA, Catalog #ab118970), following the instructions. Post 24 h X-ray irradiation, they were lysed in MDA lysis buffer using a Precellys 24 homogenizer (Bertin Instruments, Montigny-le-Bretonneux, France). The lysates were centrifuged at 13,000× *g* for 10 min at 4 °C. The supernatants (200 μL) were combined with 600 μL of thiobarbituric acid and heated at 95 °C for one hour. After cooling for 10 min, the samples were transferred to a 96-well plate and the absorbance was read at 532 nm. A standard curve was created using MDA solutions ranging from 0 to 3200 nM. Protein concentrations in the lysates were determined using a bicinchoninic acid (BCA) protein assay kit (Invitrogen, Thermo Fisher Scientific, Waltham, MA, USA), and the MDA levels were reported as nmol MDA per mg of protein.

### 2.10. Measurement of Changes in Mitochondrial Membrane Potential (MMP)

MMP changes were assessed using the JC-1 dye, a lipophilic cationic indicator (5,5′,6,6′-tetrachloro-1,1′,3,3′-tetraethylbenzimidazolylcarbocyanine iodide), purchased from Invitrogen (Waltham, MA, USA). Post 24 h X-ray irradiation, cells were collected using 0.25% trypsin-EDTA and rinsed with PBS. They were then incubated with 2 μM JC-1 dye for 15 min at 37 °C in an atmosphere containing 5% CO_2_, followed by two PBS washes. Flow cytometry was employed to analyze the stained cells, and FlowJo software (TreeStar) was used for data analysis.

### 2.11. Statistics

Statistical evaluations were conducted using the Student’s two-tailed *t*-test or one-way ANOVA with subsequent Tukey’s *post hoc* tests for multiple comparisons. All analyses were carried out using GraphPad Prism version 8.0 (GraphPad Software, San Diego, CA, USA). The results are presented as mean ± standard deviation (SD).

## 3. Results

### 3.1. Selection of Peptides from D. deserti

In *D. deserti*, the analysis of transcription start sites revealed that 85 lmRNAs start with either a 5′-ATG or a 5′-GTG sequence at the beginning of leadered mRNAs of annotated genes. Notably, the translation stop codons for these lmRNAs are found either upstream or overlapping the start codons of the annotated genes [22]. Translation of lmRNAs primarily initiates by the direct binding of a 70S ribosome monomer to the start codon [26]. Therefore, the 85 identified lmRNAs appear to represent novel translatable mRNAs coding for small proteins and peptides varying from 4 to 86 aa in length [22]. Among these, four potential peptides, named Ddes-P1 to Ddes-P4, were selected, located in the 5′-UTRs of Deide_10410, Deide_11090, Deide_19720, and Deide_23310, respectively (Table 1). These peptides were chemically synthesized (Table 1) and utilized in further experiments with DP1 and hexapeptide HP1, both noted for their substantial antioxidant properties [14]. The physicochemical properties of peptides were compared (Table 1). The net charges of the peptides varied from −3.9 to +2, with Ddes-P4 exhibiting the highest positive charge. The isoelectric points of the peptides spanned from 3.79 to 10.83, predominantly falling between 4 and 7. Most peptides demonstrated negative GRAVY scores, reflecting their hydrophilic characteristics, except for Ddes-P1 and Ddes-P3. The instability indices for most peptides exceeded 40, indicating potential instability, although DP1 was the exception, displaying a positive index.

### 3.2. Ddes-P3 Shows Antioxidant Properties In Vitro

**Figure 1:**
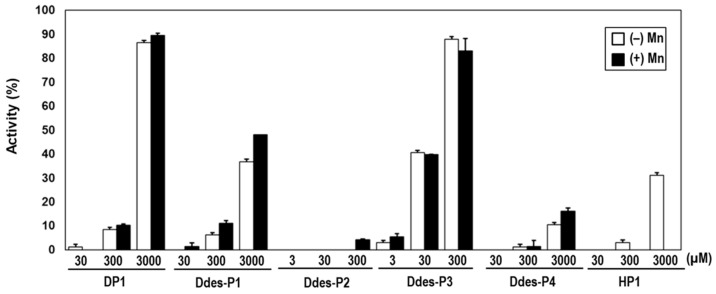
ABTS radical-scavenging activity of synthetic peptides from *D. deserti*. Peptides were tested in the presence or absence of 1 mM MnCl_2_ and incubated with the ABTS solution for 5 min. The graph shows the ABTS radical neutralization capabilities. For reference, previously identified AOPs, DP1 and HP1, were included. Values represent means ± SD from duplicate experiments.

The ABTS radical-scavenging activity assay was conducted to evaluate the antioxidant capacities of Ddes peptides. Each peptide was tested in the presence and absence of MnCl_2_ to determine its ability to neutralize ABTS radicals. The highest soluble concentrations for Ddes-P2 and Ddes-P3 were limited to 300 µM, compared to 3 mM for the other peptides (Figure 1). A dose-dependent enhancement in ABTS radical scavenging was observed across all peptides. Notably, Ddes-P3 achieved the highest antioxidant performance, neutralizing approximately 88% of ABTS radicals at 300 µM—nearly eightfold greater than that observed for DP1 at the same concentration. Despite the addition of Mn^2+^, the antioxidant properties of the peptides remained unchanged (Figure 1). CHO-K1 cells were exposed to varying concentrations of Ddes-P3 (1, 3, 10, 30, and 100 µM) for 24 h. Subsequently, cell viability was assessed using the CCK-8 assay. Results demonstrated that Ddes-P3 had no significant impact on the viability of CHO-K1 cells across all tested concentrations, suggesting a lack of cytotoxic effects (Figure S1).

### 3.3. Ddes-P3 Protects CHO-K1 Cells from Radiation by Neutralizing ROS

**Figure 2:**
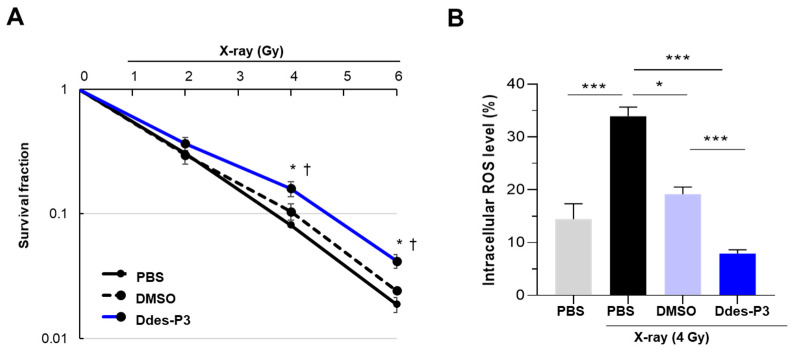
Radioprotective effects of Ddes-P3 on CHO-K1 cells. (**A**) CHO-K1 cells were pretreated with Ddes-P3 (30 µM), DMSO (0.3%, final concentration), and PBS for 2 h then subjected to radiation (2, 4, and 6 Gy). Cell survival was quantified 7 days post-irradiation using a clonogenic assay. Data points represent mean survival fractions, calculated from six replicates across three independent experiments. Statistical significance was determined using Student’s two-tailed *t*-test: * *p* < 0.05 compared to PBS-treated cells post-radiation; † *p* < 0.02 compared to DMSO-treated cells post-radiation. (**B**) Intracellular ROS production in CHO-K1 cells was analyzed by using DCHF-DA labeling. Cells underwent pretreatment with PBS, 0.3% DMSO, or 30 µM Ddes-P3 for 4 h, followed by a 4 Gy irradiation. Post-irradiation, the cells were subjected to flow cytometry analysis. The bar graph represents the mean ± SD from three independent experiments. Statistical significance was evaluated using one-way ANOVA followed by Tukey’s *post hoc* test, with * *p* < 0.05 and *** *p* < 0.001 indicating significant differences from control.

CHO-K1 cell viability was assessed using a clonogenic assay after treatment with 30 µM Ddes-P3 and subsequent irradiation. To ensure complete solubility of Ddes-P3, it was dissolved in DMSO. To account for any solvent effects, two control groups were utilized: one treated with PBS to serve as a baseline and another with 0.3% DMSO to assess potential ROS scavenging properties of the solvent itself. This dual-control setup helped ensure that observed antioxidant activities were attributed accurately to Ddes-P3. X-ray irradiation (2, 4, and 6 Gy) reduced cell survival in a dose-dependent manner across both control groups (Figure 2A). Cells treated with MP-D3 demonstrated higher survival fractions compared to those treated with PBS or DMSO. At radiation doses of 4 and 6 Gy, MP-D3-treated cells exhibited notably higher colony formation rates of 16.0% and 4.2%, respectively (Figure 2A). In contrast, DMSO-treated cells had rates of 10.4% and 2.4%, while PBS-treated cells had rates of 8.2% and 1.9% (Figure 2A). The increase in survival rates in DMSO-treated cells is attributed to the DMSO radical-scavenging effects, particularly against hydroxyl radical [27].

### 3.4. Ddes-P3 Reduces Radiation-Induced Apoptosis and Necrosis in CHO-K1 Cells

Radiation exposure results in both apoptotic and necrotic cell death, with necrosis identified as traumatic cell death that releases cellular contents into the extracellular matrix, triggering inflammatory responses in the surrounding tissues [28]. Through the Annexin V/PI assay, we found that a radiation dose of 4 Gy did not lead to considerable cell death, with about 84% of cells remaining viable (Figure S2). However, at a dose of 8 Gy, there was a significant increase in both late apoptotic and necrotic cell deaths (Figure S2). Therefore, further experiments were conducted using the 8 Gy dosage.

**Figure 3:**
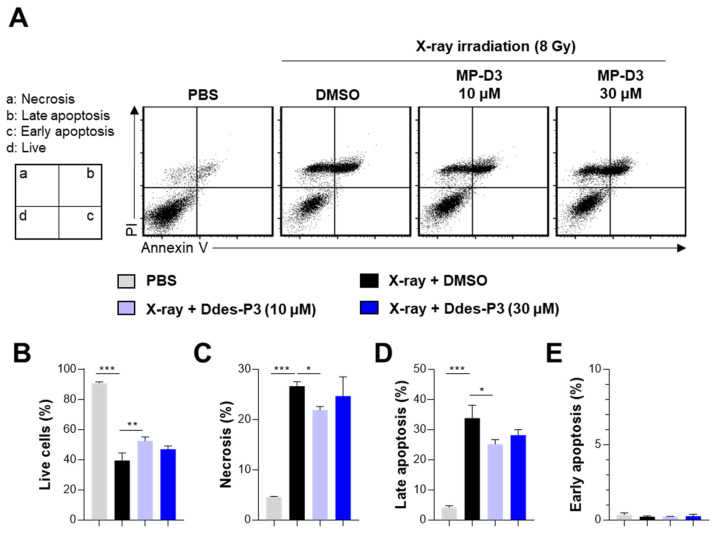
Inhibitory effects of Ddes-P3 on X-ray-induced apoptotic and necrotic cell death. CHO-K1 cells were pretreated with 10 or 30 µM Ddes-P3 for 3 h before X-ray irradiation. Non- and irradiated groups without Ddes-P3 were treated with PBS and 0.3% DMSO, respectively. After 24 h of X-ray irradiation, the cells were stained with an Annexin V/PI apoptosis detection kit and analyzed by flow cytometry. (**A**) Representative dot plot images from each group. (**B**) Live cells gated as Annexin V^−^PI^−^ cells. (**C**) Necrotic cells gated as Annexin V^−^PI^+^ cells. (**D**) Late apoptotic cells gated as Annexin V^+^PI^+^ cells. (**E**) Early apoptotic cells gated as Annexin V^+^PI^−^ cells. Bar graphs are expressed as mean ± SD of three independent experiments. Statistics were analyzed using one-way ANOVA with Tukey’s *post hoc* test. * *p* < 0.05, ** *p* < 0.01, or *** *p* < 0.001.

We examined the protective effects of Ddes-P3 against X-ray-induced apoptosis and necrosis in CHO-K1 cells. X-ray irradiation at a dose of 8 Gy significantly reduced the live cell population, with a corresponding increase in necrotic and late apoptotic cell death (Figure 3A–D). Interestingly, X-ray exposure did not significantly alter early apoptosis (Figure 3E). Pretreatment with Ddes-P3 at 10 μM substantially enhanced live cell recovery (Figure 3B), which was associated with a decrease in both necrotic and late apoptotic cell populations (Figure 3C,D). These findings underscore the potential of Ddes-P3 to mitigate radiation-induced cell death through its protective mechanisms.

### 3.5. Ddes-P3 Reduces Cellular Damage in Irradiated CHO-K1 Cells

**Figure 4:**
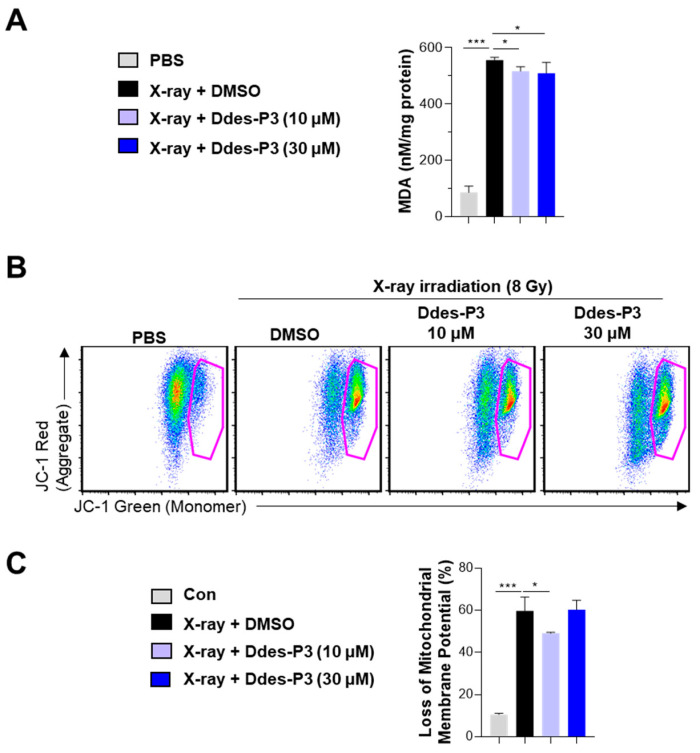
Inhibitory effects of Ddes-P3 on X-ray-induced MDA accumulation and MMP loss. CHO-K1 cells were pretreated with 10 or 30 µM Ddes-P3 for 3 h before X-ray irradiation. Non- and irradiated groups without Ddes-P3 were treated with PBS and 0.3% DMSO, respectively. (**A**) After 24 h of X-ray irradiation, three wells in each group were pooled, and the cells were homogenized with MDA lysis buffer. Level of MDA in cell lysates were measured by colorimetric MDA assay kit. (**B**,**C**) The cells were harvested by 0.25% trypsin-EDTA after 24 h of irradiation and stained with JC-1 dye for 15 min at 37 °C in a 5% CO_2_. The stained cells were analyzed by flow cytometry. (**B**) The representative dot plot images from each group. The pink lines outline the populations exhibiting mitochondrial membrane depolarization, indicative of mitochondrial damage. (**C**) The bar graph showing the percentage of MMP loss, expressed as mean ± SD of three independent experiments. Statistics were analyzed using one-way ANOVA with Tukey’s *post hoc* test. * *p* < 0.05 or *** *p* < 0.001.

During cellular damage caused by radiation exposure, various cellular organelles undergo lipid peroxidation, including the cell membrane, mitochondria, and endoplasmic reticulum [7]. The hydrophobic amino acids in AOPs enhance their solubility in lipids, aligning their antioxidant activity with processes like lipid peroxidation [4]. Given the significant hydrophobic nature of Ddes-P3 (Table 1), we opted to measure malondialdehyde (MDA) levels, a primary biomarker of lipid peroxidation [29], to evaluate Ddes-P3′s efficacy in inhibiting lipid peroxidation. As shown in Figure 4A, X-ray irradiation notably increased MDA levels in CHO-K1 cells, signifying increased oxidative stress. Conversely, treatment with Ddes-P3 significantly reduced MDA production (Figure 4A), demonstrating its capacity to protect against oxidative-stress-induced lipid peroxidation.

Mitochondria regulate apoptosis and play an essential role in ROS generation during oxidative phosphorylation. Radiation exposure triggers oxidative stress, increasing ROS production from water radiolysis and enhancing mitochondrial ROS accumulation, thereby impairing mitochondrial function [30,31]. We explored the protective effects of Ddes-P3 on mitochondrial function using JC-1, a cationic dye that detects changes in mitochondrial membrane potential (MMP). In the control group, we observed high red fluorescence, indicating the accumulation of JC-1 dye in healthy mitochondria forming J-aggregates (Figure 4B). In contrast, X-ray irradiation led to high green fluorescence, which means that JC-1 dye remained in its monomeric form due to its inability to penetrate damaged mitochondria (Figure 4B). This increase in green fluorescence suggests a significant loss of MMP in the irradiated group. Treatment with 10 µM Ddes-P3 significantly reduced the presence of JC-1 monomers and decreased the percentage of MMP loss (Figure 4B,C). These observations suggest that Ddes-P3 effectively mitigates radiation-induced mitochondrial dysfunction and cellular damage by preserving mitochondrial membrane potential, highlighting its potential as a radioprotective agent.

## 4. Discussion

Glutathione (GSH), a tripeptide made of glutamine, cysteine, and glycine, is a crucial antioxidant in bacterial cells. It protects bacteria from oxidative stress by neutralizing ROS and maintaining cellular redox balance [32]. In addition to GSH, bacteria generate other peptides with antioxidant properties. Notably, cyclic dipeptides like cyclo (His-Pro) and cyclo (d-Tyr-d-Phe), derived from *Bacillus* species, are known for their effective radical-scavenging activities [33,34]. Additionally, peptides that combine antimicrobial and antioxidant actions have been discovered [35]. Given that many bacteria produce antimicrobial peptides (AMPs), these organisms are potential sources of antioxidant AMPs. For instance, the AMP YD1 (APKGVQGPNG) from *B. amyloliquefaciens* is recognized for its potent antioxidant effects [36]. Consequently, bacteria are capable of producing a wide range of endogenous peptides that fulfill multiple biological roles, including significant contributions to antioxidant defense mechanisms [5].

Recent advancements in large-scale transcriptome analyses have effectively characterized lmRNAs in bacteria and archaea, which can initiate translation directly at TSSs [20]. These leaderless genes vary significantly across genomes, with a prevalence ranging from 3.3% to 53.6% [37]. This variation suggests that a substantial proportion of proteins are synthesized from lmRNAs in some species. For example, in *Mycobacterium tuberculosis*, 22% of the 2524 genes transcribed during the exponential phase are expressed as lmRNAs [38]. A comprehensive analysis of protein and mRNA levels in this bacterium shows that the presence or absence of a leader sequence in mRNA does not necessarily affect translation efficiency [39]. In addition, genome-wide mapping of *M. tuberculosis* has identified 317 lmRNAs originating from novel ORFs, with 197 encoding peptides ranging from 5 to 50 amino acids [40]. Similarly, in *D. deserti*, approximately 39% of 173 novel leaderless transcripts are predicted to code for peptides of 4 to 15 residues [22]. These short leaderless transcripts can be potential templates for synthesizing peptides. Leaderless genes are notably prevalent in *Deinococcus*, with in silico analyses showing that 46.1% of the genes in the *D. radiodurans* genome are leaderless [21]. This significant occurrence of leaderless genes may be linked to their distinct traits, including their exceptional resistance to radiation. The discovery of peptide-centric antioxidant complexes in protein-free extracts from *D. radiodurans* highlights its role as a principal source of antioxidant peptides [11]. Therefore, exploring the genomes of radiation-resistant bacteria such as *Deinococcus* for short lmRNAs ranging from 15 to 45 nucleotides could pave the way for discovering new AOPs.

While the structure–activity relationships of AOPs have been studied, focusing on how their molecular structure—including MW, aa composition, sequence, secondary structure, and hydrophobicity—affects their antioxidant activity, the underlying mechanisms that define these relationships are not fully understood. Most natural AOPs comprise 2 to 10 amino acids and typically have an MW under 1000 Da [41]. This aligns with data from the Antioxidant Database (AODB), which catalogs 1380 AOPs, excluding monopeptides [42]. These peptides range from 2 to 68 residues, with MWs between approximately 210 Da and 7440 Da. Notably, peptides containing 2 to 10 amino acids comprise 88.2% of the total. Ddes-P3 is an AOP composed of 10 amino acids with an MW of 1106 Da (Table 1).

The activity of an AOP is affected not just by the length of its peptide chain but more significantly by its aa composition. Peptides with robust antioxidant properties usually exhibit a greater presence of hydrophobic amino acids [4]. Analysis of 183 peptides, each with 9 to 11 residues retrieved from the AODB (Table S1), shows that Pro (P) and Gly (G) are the most prevalent, comprising 20.7% of the total amino acids (Figure S3). Following these are aliphatic and hydrophobic amino acids like Leu (L), Ala (A), and Val (V), which make up 24.9%. Conversely, other nonpolar hydrophobic amino acids like Phe (F), Trp (W), Met (M), and Cys (C) are less common, representing just 7.6% of the amino acids in these peptides (Figure S3). Despite Ddes-P3 having a lower GRAVY index than Ddes-P1, it uniquely lacks hydrophilic amino acids such as Gln (Q), Asn (N), Glu (E), Asp (D), Arg (R), and Lys (K)—an attribute not found in other tested peptides (Table 1). Additionally, Ddes-P3 features a sequence of three consecutive hydrophobic amino acids, MMW, at its N-terminus. The sequence and positioning of these amino acids are critical for enhancing antioxidant activities, with the hydrophobic nature at the N-terminal playing a crucial role in preventing lipid peroxidation [41,43]. This is achieved by improving the peptide’s solubility in lipid environments and accessibility to fat-soluble ROS and polyunsaturated fatty acids [44]. In this study, Ddes-P3 has effectively inhibited lipid peroxidation and preserved mitochondrial membrane integrity (Figure 4).

In our study, cell survival rates and mitochondrial membrane potential depolarization were both negatively impacted in the high-concentration Ddes-P3 (30 μM) group compared to the low-concentration (10 μM) group (Figure 3 and Figure 4), which can be attributed to the biphasic nature often observed with antioxidant compounds. While low concentrations of antioxidants primarily scavenge ROS, higher concentrations might lead to pro-oxidant effects depending on their chemical structure and the surrounding conditions [45,46]. Upon scavenging ROS, antioxidants can transform into their oxidized forms, potentially exacerbating oxidative stress or causing DNA damage [47]. For Ddes-P3, this is particularly critical as its methionine residues are prone to oxidation, forming methionine sulfoxide. Additionally, the tryptophan residues could oxidize into hydroxytryptophan or kynurenine, fostering an oxidative environment [48,49]. This dual nature of antioxidant and pro-oxidant activity becomes more pronounced under conditions of radiation exposure, disrupting the balance between protective and damaging effects. Further research is warranted to optimize the concentration of Ddes-P3 to maximize protection without inducing further oxidative damage.

Radioprotectors serve as agents that protect organisms from cellular and molecular damage during irradiation, mainly by bolstering antioxidant defenses to neutralize free radicals. A variety of molecules, including sulfhydryl compounds, vitamins, dietary antioxidants, phytochemicals, and extracts from plants and bacteria, have demonstrated radioprotective capabilities [50]. Peptides, in particular, have emerged as highly appealing radioprotectors due to their feasible production methods and optimal pharmacological traits—such as nontoxicity, specificity, small molecular size, minimal immunogenicity, rapid diffusion, and effective distribution [51]. Numerous peptides, including the linear tetrapeptide EA230, casein phosphopeptides, walnut oligopeptides, scorpion venom peptide B5, and honeybee venom melittin, along with DP1 and HP1 used in this study, are recognized for their radioprotective properties, acting as antioxidants and free radical scavenger properties [14,15,51].

Mitochondria-targeted antioxidant peptides are being investigated due to the high ROS levels to which mitochondria are exposed [52,53]. Szeto-Schiller (SS) peptides, consisting of two positively charged and two aromatic amino acids, effectively scavenge free radicals, diminish ROS in mitochondria, and prevent the translocation of the mitochondrial membrane and release of cytochrome C [52,54]. The XJB series of peptide-based antioxidants, including XJB-5-125, XJB-5-131, and XJB-5-197, are designed to combat mitochondrial oxidative stress [53]. These peptides incorporate a structure derived from the cyclopeptide antibiotic Gramicidin S, which targets mitochondrial membranes. The antioxidant nitroxide, specifically 2,2,6,6-tetramethyl piperidine-1-oxyl (TEMPO), is used to effectively neutralize reactive oxygen species (ROS). A leucine-rich peptide backbone functions as a bridge, connecting the Gramicidin-S-derived scaffold and TEMPO, facilitating the direct targeting of mitochondrial membranes [55]. Notably, pretreatment with 20 μM XJB-5-125 increased clonogenic survival of mouse embryonic cells subjected to 4 Gy of IR and significantly reduced delayed apoptosis in cells exposed to 10 Gy [56]. Clonogenic assays also showed that 10 μM of XJB-5-131 and its truncated analog JP4-039, featuring a shortened alkene dipeptide linked to TEMPO, effectively protected human umbilical cord blood progenitor cells across 0–8 Gy of IR [57]. Similarly, Ddes-P3 at 10 μM reduced MDA production, preserved mitochondrial membrane potential, and prevented apoptosis, demonstrating radioprotective capacities comparable to those of XJB-5-131 and positioning it as a promising candidate for radioprotective applications.

## 5. Conclusions

*Deinococcus* is renowned for its robust antioxidant repertoire, including compounds such as the carotenoid deinoxanthin and the exopolysaccharide DeinoPol, which provide significant radioprotective and antioxidant properties [58]. These molecules establish *Deinococcus* as a reservoir for bioactive compounds useful for radiation protection. In this study, we focused on the antioxidant peptide Ddes-P3, derived from *D. deserti*. Our findings highlighted Ddes-P3’s ability to mitigate oxidative stress and enhance cellular resilience under irradiative conditions. It excels in scavenging ROS and safeguarding cellular constituents from radiation-induced damage, positioning it as an effective, nontoxic radioprotector suitable for biotechnological and therapeutic applications.

Moreover, our research emphasizes the role of lmRNA in the synthesis of bioactive peptides within radiation-resistant bacteria like *Deinococcus*. The involvement of lmRNA paves the way for future investigations aimed at utilizing these genetic elements to develop novel AOPs that could play a crucial role in enhancing cellular resistance to oxidative stresses. The promising results with Ddes-P3 underscore the potential of mitochondria-targeted antioxidant peptides in radioprotection. Future efforts will be dedicated to optimizing and characterizing Ddes-P3 alongside established mitochondria-targeting peptides such as XJB-5-131. These studies are aimed at systematically evaluating their protective effects against radiation-induced cellular damage, thereby potentially introducing novel therapeutic strategies for managing oxidative stress.

## Figures and Tables

**Table 1:** Physicochemical characteristics of peptides.

Peptide Name	Sequence (No. ofaa)	Predicted pI/MW	Net Charge at pH 7.0	Instability Index	GRAVY Index	Water Solubility
Ddes-P1	MPFEEITLGAA (11)	3.79/1178.37	−2	105.97 (unstable)	0.627 (hydrophobic)	poor
Ddes-P2	VSHNEQQMEEE (11)	4.09/1359.39	−3.9	124.91 (unstable)	−2.036 (hydrophilic)	good
Ddes-P3	MMWSSGHISA (10)	6.49/1106.28	0.1	69.32 (unstable)	0.320 (hydrophobic)	poor
Ddes-P4	MSVTKERRL (9)	10.83/1119.35	2	111.92 (unstable)	−0.889 (hydrophilic)	good
DP1	DEHGTAVMLK (10)	5.32/1100.25	−0.9	−25.89 (stable)	−0.350 (hydrophilic)	good
HP1	HMHMHM (6)	7.02/823.02	0.3	199.27 (unstable)	−0.650 (hydrophilic)	poor

## Data Availability

The original contributions presented in the study are included in the article and Supplementary Materials, further inquiries can be directed to the corresponding author.

## Supplementary Materials

The following supporting information can be downloaded at: https://www.mdpi.com/article/10.3390/microorganisms12112161/s1, Figure S1: Cytotoxicity assessment of Ddes-P3 on CHO-K1 cells; Figure S2: Flow cytometry analysis of CHO-K1 cell death post-irradiation; Figure S3: Distribution of amino acids in 183 antioxidant peptides; Table S1: Antioxidant peptides consisting of 9 to 11 amino acids cataloged in the antioxidant database (AODB).
