# Supplementary material for: A Novel Deinococcus Antioxidant Peptide Mitigates Oxidative Stress in Irradiated CHO-K1 Cells
Source: Microorganisms. 2024 Oct 26;12(11):2161. doi: 10.3390/microorganisms12112161 (PMC11596967; doi:10.3390/microorganisms12112161)
Supplement: Supplementary file 1 [file microorganisms-12-02161-s001.zip › Supplementary Figure.pdf]

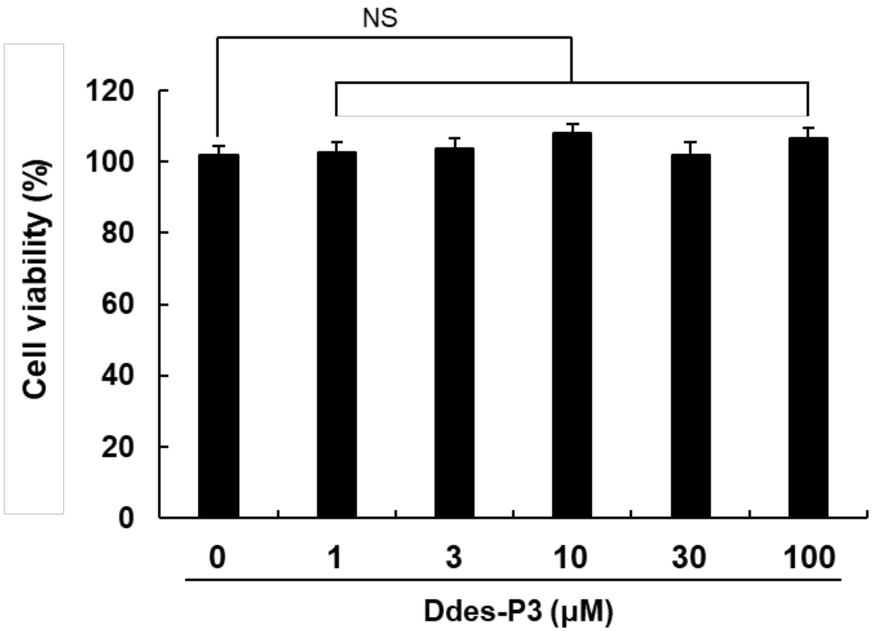

Figure S1. Cytotoxicity assessment of Ddes-P3 on CHO-K1 Cells. Cytotoxic effects of Ddes-P3 were evaluated on CHO-K1 cells exposed to concentrations ranging from 1 to 100 μM for 24 hours at 37°C. Cell viability was quantified using the CCK-8 assay. The graph presents mean cell viability percentages ± standard deviation (SD) derived from triplicate samples. Statistical differences were assessed by Student's two-tailed *t-test*, indicating no significant difference (NS) compared to the control group without treatment.

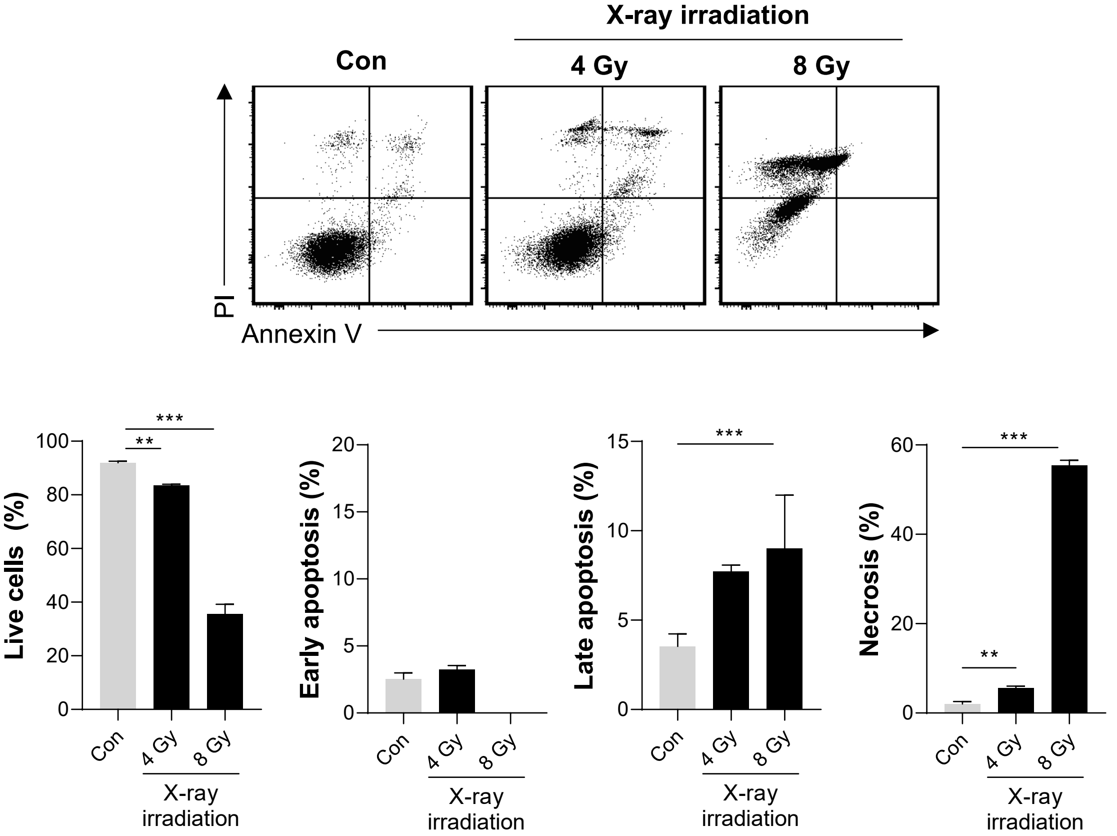

Figures S2. Flow cytometry analysis of CHO-K1 cell death post-irradiation. CHO-K1 cells were exposed to doses of 4 or 8 Gy of X-ray irradiation. After 24 hours, the cells were stained with an Annexin V/PI apoptosis detection kit and analyzed by flow cytometry. The dot plot images represent the distribution of cell populations: live cells (Annexin V^{-}PI^{-}) in the lower left quadrant, necrotic cells (Annexin V^{-}PI^{+}) in the upper left, early apoptotic cells (Annexin V^{+}PI^{-}) in the lower right, and late apoptotic cells (Annexin V^{+}PI^{+}) in the upper right. Bar graphs reflect the percentage of each cell population, with mean ± SD of three independent experiments. Statistics were analyzed using one-way ANOVA with Tukey's *post-hoc* test. $^{**}p < 0.01$ or $^{***}p < 0.001$

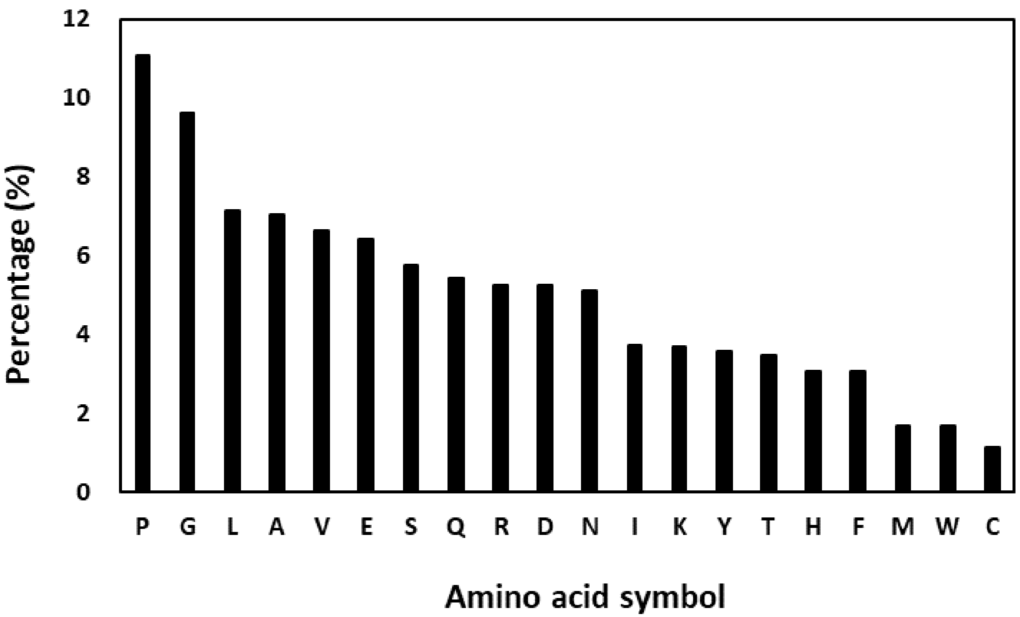

Figure S3. Distribution of amino acids in 183 antioxidant peptides. The bar graph shows the relative abundance of each amino acid in a dataset of 183 antioxidant peptides, each containing between 9 and 11 residues, extracted from the AODB (Table S1). The percentages represent the occurrence of each amino acid, calculated by dividing the number of specific residues by the total number of amino acids (1,820) present in the dataset.
